# Identification of fine antigenic epitopes of Tamdy virus glycoprotein Gn fragment and establishment of ELISA detection method

**DOI:** 10.1186/s13071-024-06646-2

**Published:** 2025-01-24

**Authors:** Yujiao Fu, Liping Liu, Beibei Zhang, Xiaoshan Chao, Junxia Jin, Ying Wang, Juntao Ding

**Affiliations:** 1 College of Life Science and Technology, Xinjiang University, Urumqi, 830017 China; 2 Xinjiang Key Laboratory of Biological Resources and Genetic Engineering, Urumqi, 830017 China; 3 College of Textiles and Clothing, Xinjiang University, Urumqi, 830017 China

**Keywords:** Tamdy virus, Glycoprotein Gn, ELISA, Antigenic epitopes, Western blotting

## Abstract

**Background:**

Tamdy virus (TAMV) was first isolated in Uzbekistan and Turkmenistan. In 2018, it was found in China, marking its entry into the molecular research era. TAMV is linked to febrile diseases, but its epidemiology and spillover risks are poorly understood, necessitating urgent molecular research and detection method development.

**Methods:**

The secondary structure of TAMV glycoprotein Gn was predicted, and the results showed that it had rich antigenic epitopes. According to the predicted results, glycoprotein Gn was divided into 46 truncated 16-peptides by modified synthetic peptide method, and the antigenicity of 46 truncated 16-peptides was verified by western blotting analysis.

**Results:**

The results showed that P8, P9, P24, P25, P28, P29, and P39 had antigenicity. Subsequently, the seven positive 16-peptide sequences with antigenicity were truncated to form 8-peptide sequences with an overlap of seven amino acids. After analysis with the same method, eight fine antigenic epitopes E1 (^58^VINSTLDH^65^), E2 (^65^HVGSWGMP^72^), E3 (^68^SWGMPVTT^75^), E4 (^187^IRNQPFKS^194^), E5 (^195^FNVEVQ^200^), E6 (^226^AVVEHH^231^), E7 (^228^VEHHGNKA^235^), and E8 (^310^RGGRR^314^) were identified, all of which were located on the three-dimensional surface of glycoprotein Gn and were highly conserved in different TAMV strains.

**Conclusions:**

Eight precise epitopes were identified, and an indirect ELISA method based on fusion multiepitope peptide (r-Gn-MEPX_2_) was developed and implemented, featuring high sensitivity, accuracy, and specificity.

**Graphical Abstract:**

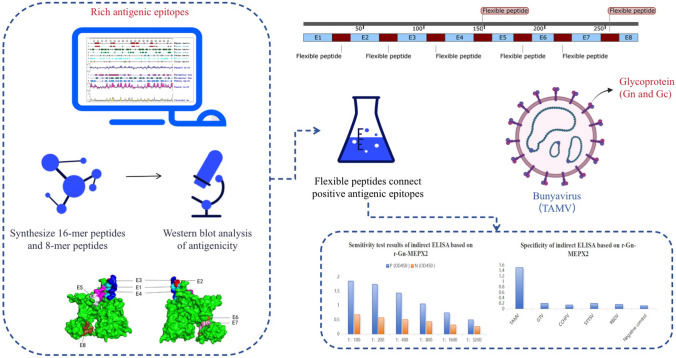

## Background

Ticks are distributed globally and are parasites that exert an impact on human and animal life [1, 2]. They typically carry bacteria [3], viruses [4], and protozoan pathogens [5] and transmit them to humans and animals. Tick bites can result in skin lesions, papules, and intense itching [6].

In recent years, due to global climate change, ecosystem changes, and frequent communication at home and abroad, the risk of the spread and spillover of tick-borne diseases has gradually escalated [7], and the diagnosis and treatment of tick-borne diseases are generally ambiguous. The diagnosis process is often based on clinical judgment in conjunction with laboratory testing, treatments may require disease-dependent approaches, and co-infections complicate or increase the severity of the clinical picture [8], so the research on tick-borne viruses is of great significance.

Tamdy virus (TAMV) pertains to the *Bunyavirales* (genus *Orthonairovirus*, family *Nairoviridae*) [9]. *Bunyavirales* virus encompasses nine families of negative-stranded RNA viruses [10]. The genome is segmented into three segments: small (S) and large (L) fragments encode proteins implicated in genome replication in the infected cytoplasm, and the middle (M) segment encodes viral glycoproteins Gn and Gc [11, 12], which enter the cell via receptor-mediated endocytosis [13]. Most *Bunyavirales* viruses will inflict harm upon humans and animals, such as fever, hemorrhagic fever, ocular disease, and neurologic disease [14–16], and studies have indicated that, in recent years, their effects on plants have also been expanding (mainly orthospoviruses (*Tospoviridae*), tenuiviruses (*Phenuiviridae*), and emaraviruses (*Fimoviridae*)) [17]. Therefore, the prevention and control of these viruses is very important.

From 1971 to 1974, Lvov D. K. et al. initially isolated TAMV from *Hyalomma asiaticum* in Uzbekistan and Turkmenistan [18]. In May 2018, this virus was isolated in China for the first time, and the strain was designated as XJ01/TAMV/China/2018 [19]. The complete genomic coding sequence of TAMV was obtained for the first time in 2016 [20], signifying the entry of research on this virus into the molecular era.

Although there have been no patients with confirmed TAMV, serological studies have revealed that there was a small-scale outbreak of human TAMV infection in northwest China in 2007 [21]. Current studies have indicated that TAMV is a pathogen related to human febrile diseases [22], but its epidemiology and the risk of TAMV spillover are still poorly understood. Thus, molecular research on TAMV and the establishment of detection methods are urgently needed.

## Methods

### Sequence design of 16-peptide and 8-peptide truncated by TAMV-Gn

To identify the minimum antigenic epitope motif of TAMV-Gn, TAMV-Gn (GenBank accession number: MT815990) was designed into 49 overlapping 8-amino-acid 16-peptides by employing the improved biosynthetic peptide strategy, and then each positive 16-peptide was truncated into 9 overlapping 7-amino-acid 8-peptides. Restriction sites for *Bam*H I and *Sal* I were introduced at both ends of the sequence, and the synthesis was accomplished by Shanghai Saibaisheng Gene Technology Co., Ltd.

### Construction and prokaryotic expression of truncated 16-peptide and 8-peptide recombinant plasmid

PXXGST-3 was digested by *Bam*H I and *Sal* I. After gel recovery, the truncated 16-peptide and 8-peptide fragments were ligated and transformed into *E. coli* BL21 (DE3) competent cells. The cultured bacteria were thermally induced and identified by SDS-PAGE and sent to Shanghai for sequencing.

### Western blotting analysis of truncated 16-peptide and 8-peptide recombinant plasmids

The successfully induced protein samples were analyzed by western blotting with rabbit anti-Gn protein polyclonal antibody (1:2000), and the binding of the antibody to the target protein was observed in the chemiluminescence imager.

### Conservation analysis and three-dimensional conformational localization of fine epitopes of TAMV glycoprotein Gn

The three-dimensional structure of TAMV glycoprotein Gn was presented by Python 2.7 software, and the fine epitopes were located on its surface. The amino acid sequences of glycoprotein Gn of different TAMV strains were compared in GeneDoc software, and the conservation of the epitope motifs identified in this study was analyzed among different TAMV strains.

### Verification of antigenicity of minimal antigenic epitope by TAMV-positive sheep serum

The antigenicity of positive 16-peptides and eight B-cell epitopes (BCEs) was verified through western blotting. The TAMV IgG-positive sheep serum was employed as the first antibody (1:200), and the HRP-rabbit anti goat IgG (1:5000) was utilized as the second antibody.

### Design of multiepitope peptides

The selected fine antigenic epitopes were tandem with flexible peptides and double copied into MEPX_2_. The fragments were optimized and sent to Shanghai Genray Biotech Co., Ltd for synthesis.

### Construction of recombinant plasmid pET-28a-Gn-MEPX_2_

The gene sequence of Gn-MEPX_2_ was ligated into the pET-28a plasmid digested by *Bam*H I and *Sal* I and then transformed into *E. coli* Rosetta (DE3) competent cells. It was identified by double enzyme digestion and sent to Shanghai Shenggong Bioengineering Co., Ltd for sequencing.

### Induced expression and purification of fusion multiepitope peptide (r-Gn-MEPX_2_)

The r-Gn-MEPX_2_ was transformed into the competent cells of *E. coli* Rosetta (DE3), then smeared and cultured. The cultured bacteria were induced and the cells were broken by ultrasonic fragmentation. The expression and localization of r-Gn-MEPX_2_ were determined by SDS-PAGE.

The precipitate formed after ultrasound was dissolved, denatured, and eluted with imidazole. The eluate with better performance was dialyzed and concentrated by ultrafiltration. The protein concentration was determined, and the samples were then stored at −80 ℃.

### Identification of antigenicity of fusion multiepitope peptide (r-Gn-MEPX_2_) by western blotting

The purified r-Gn-MEPX_2_ was analyzed by western blotting with the use of rabbit anti-Gn protein polyclonal antibody (1:10,000) and mouse anti-His monoclonal antibody (1:5000). The binding of the antibody to the target protein was observed by chemiluminescence imager.

### Optimization of working conditions of indirect ELISA detection method based on r-Gn-MEPX_2_

The working method was optimized by choosing different antigen concentrations, primary antibody dilution ratios, primary antibody incubation times, secondary antibody dilution ratios, secondary antibody incubation times, and chromogenic times. Finally, the absorbance at 450 nm was determined by enzyme labeling instrument. P/N > 2.1 was regarded as positive.

### Verification of working conditions of indirect ELISA detection method based on r-Gn-MEPX_2_

1. Accuracy test: The natural animal sera were screened by this method. The positive and negative sera were used as the first antibody, and the purified r-Gn-MEPX_2_ protein was used as the antigen, which was verified by western blotting.
2. Sensitivity test: Using the purified r-Gn-MEPX_2_ protein as antigen, TAMV-positive and TAMV-negative sera were diluted from 1:100 to 1:3200. The sensitivity was evaluated according to the established indirect ELISA detection method.
3. Specificity test: SFTSV (Severe fever with thrombocytopenia syndrome virus)-, GTV (Guertu virus)-, RBDV (Rhabdo virus)-, and CCHFV (Crimean-Congo hemorrhagic fever virus)-related polyclonal antisera and TAMV-positive and TAMV-negative sera preserved in the laboratory were used as the first antibody, and the established indirect ELISA assay was used to evaluate the specificity of the method.
4. Conformity test: Using the natural animal serum detected by immunofluorescence in Vero cells infected by TAMV whole virus as the first antibody, the established indirect ELISA detection method was used to determine the coincidence rate of the indirect ELISA detection method.

### Preliminary application of indirect ELISA detection method based on r-Gn-MEPX_2_

To understand the epidemic situation of TAMV in some areas of Xinjiang, TAMV IgG was detected in 327 animal sera collected from Xinjiang. Among them, there were 87 sera from Burjin sheep in 2019, 25 sera from Tashkurgan shepherds in 2016, 30 sera from Shawan shepherds in 2015, 90 sera from hot spring marmots in 2014, 30 sera from Manas’s camels in 2014, and 65 sera from *Spermophilus undulatus* in Yiwu County in 2014.

## Results

### Induced expression and antigenicity identification of TAMV-Gn truncated 16-peptide

**Fig. 1:**
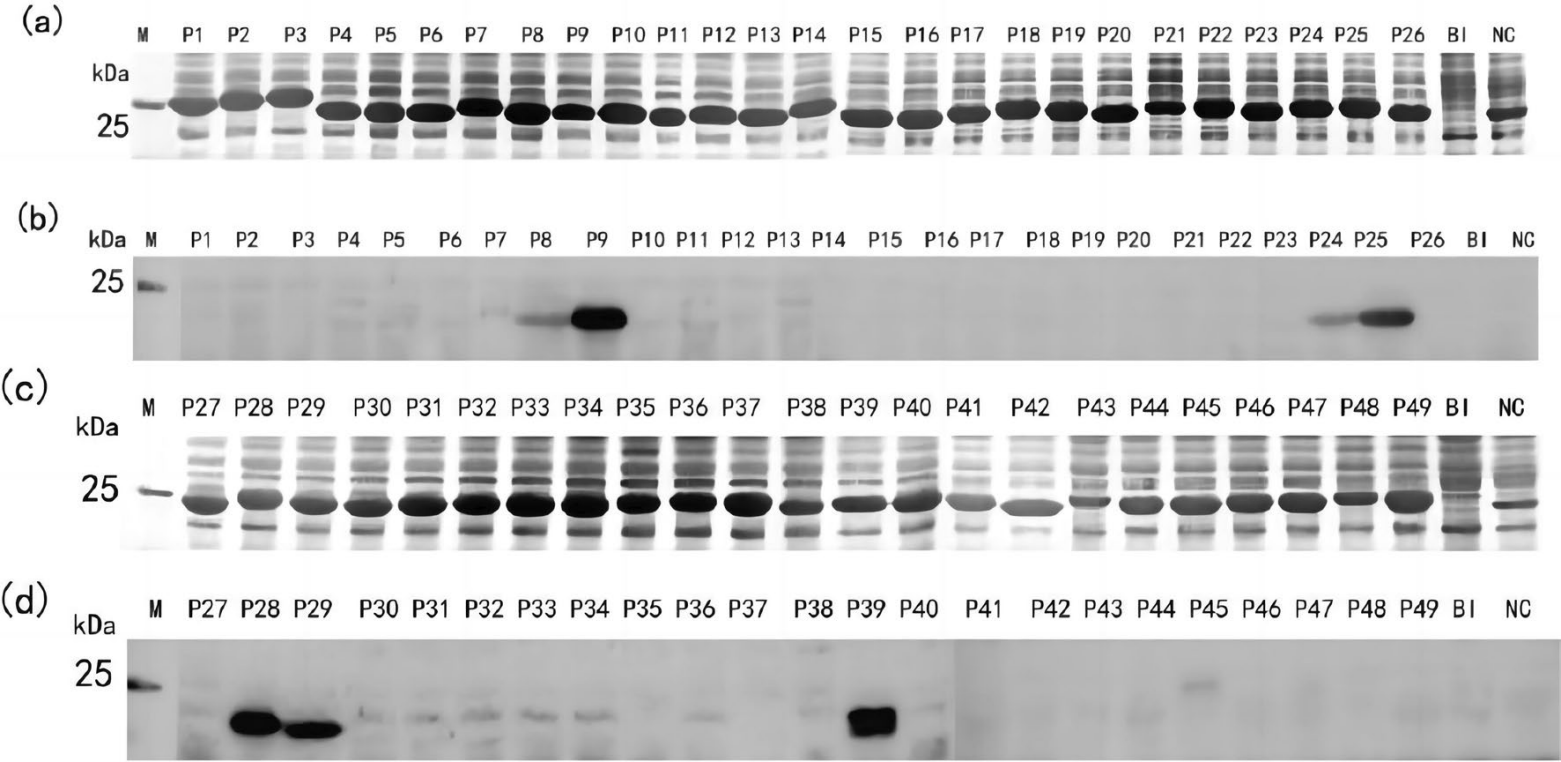
Results of SDS-PAGE and western blotting antigenicity of glycoprotein Gn truncated 16-peptides P1–P49 induced expression. (**a**, **c**) SDS-PAGE identification of 49 fused GST label truncated 16-peptides induced expression. (**b**, **d**) Antigenicity detection results of 49 GST labeled 16-peptides western blotting. *BI* total protein before induction, *NC* negative control (GST-188 label protein expressed by pXXGST-3 vector), *M* protein marker

Gn was divided into 49 truncated 16-peptides and expressed in the pXXGST-3 vector. SDS-PAGE results indicated that a fusion protein of approximately 23 kDa could be observed (Fig. 1). Western blotting results demonstrated that P8, P9, P24, P25, P28, P29, and P39 were recognized by rabbit anti-Gn protein polyclonal antibodies.

### Induced expression and antigenicity identification of TAMV-Gn truncated octapeptide

To further identify the minimum epitope motif, the positive 16-peptides P8, P9, P24, P25, P28, P29, and P39 were truncated into 8-peptides, and the prokaryotic expression vector pXXGST-3 was constructed.

**Fig. 2:**
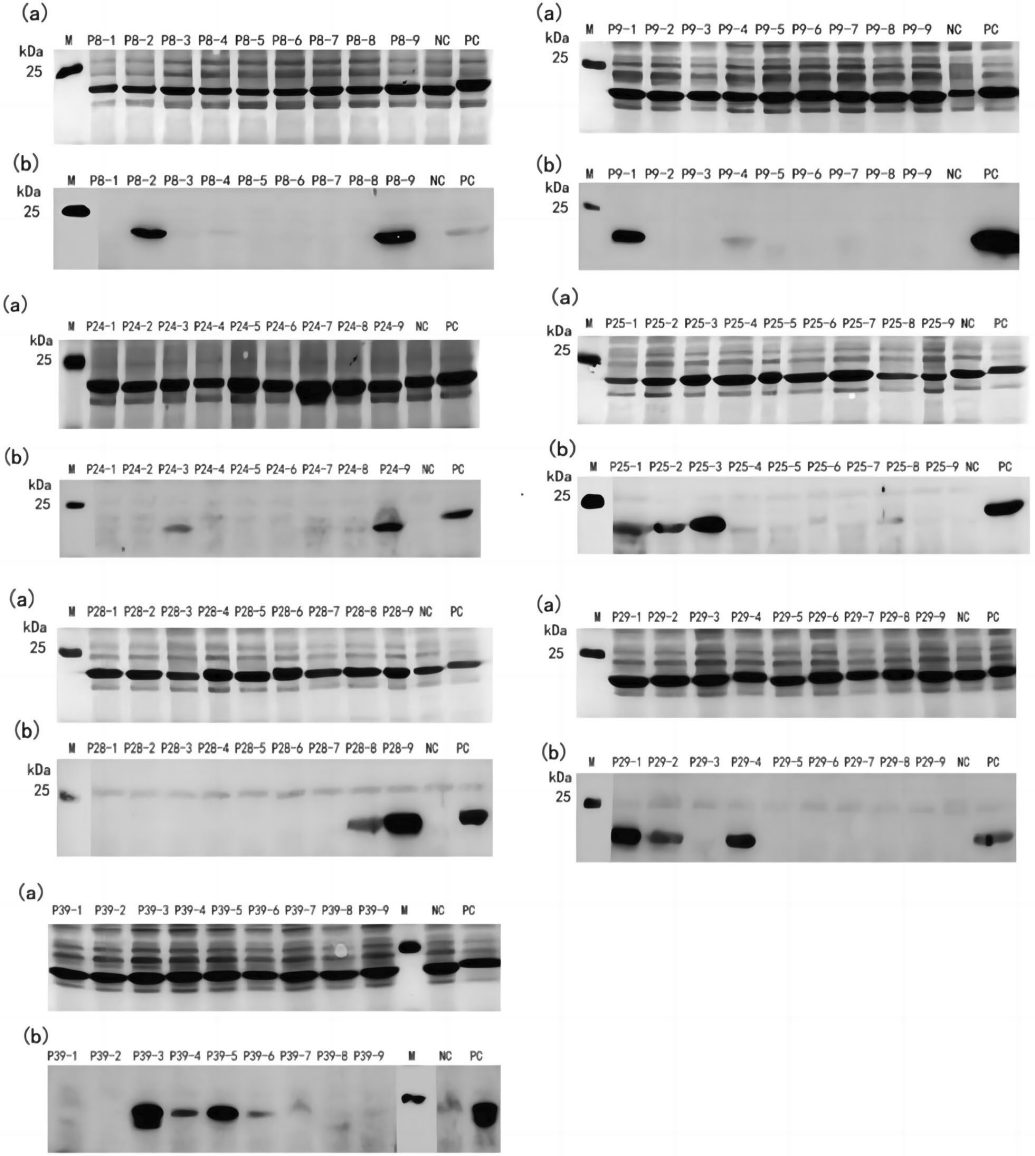
Identification of truncated octapeptide epitopes. **a** Truncated SDS-PAGE results of octapeptide expression. **b** Truncated octapeptide western blotting antigenicity test results. **c** Truncated octapeptide minimum epitope sequence mapping results. *NC* negative control (GST-188 tagged protein expressed by pXXGST-3 vector), *PC* positive 16-peptide, *M* protein marker

The results indicated that truncated octapeptide was expressed correctly and a fusion protein with a size of about 22 kDa could be observed. Western blotting results demonstrated that P8-2, P8-9, P9-1 (P8-9), P9-4, P24-3, P24-9, P25-1 (P24-9), P25-2, P25-3, P28-8, P28-9, P29-1 (P28-9), P29-2, P29-4, P39-3, P39-4, P39-5, and P39-6 (Fig. 2) could be recognized by rabbit anti-Gn protein polyclonal antibodies. The smallest epitope motifs are ^58^VINSTLDH^65^ (named E1), ^65^HVGSWGMP^72^ (named E2), ^68^SWGMPVTT^75^ (named E3), ^187^IRNQPFKS^194^ (named E4), ^195^FNVEVQ^200^ (named E5), ^226^AVVEHH^231^ (named E6), ^228^VEHHGNKA^235^ (named E7), and ^310^RGGRR^314^ (named E8).

### Distribution of minimal antigenic epitope motif on the three-dimensional structure of TAMV glycoprotein Gn

**Fig. 3:**
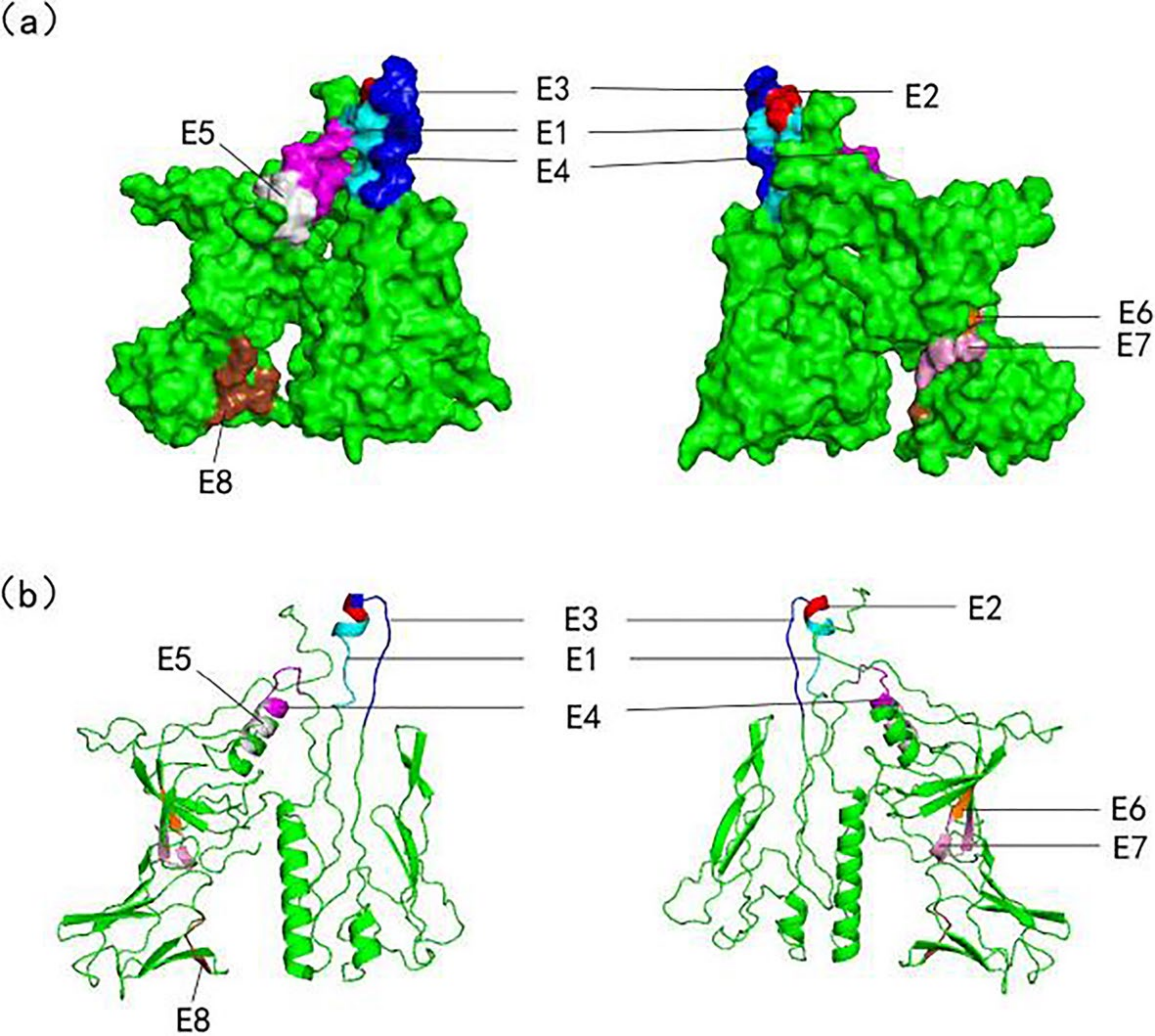
Distribution of the minimal antigen epitope motif on the three-dimensional structure of TAMV glycoprotein Gn. **a** Results of surface distribution form display. **b** The results of the presentation in cartoon form. The molecular surfaces of the eight minimum epitopes are shown in different colors (E1, cyan; E2, red; E3, blue; E4, purplish red; E5, gray; E6, orange; E7, pink; E8, brown)

I-TASSER online homology modeling, Pymol™ software simulates the three-dimensional structure of TAMV Gn, locates the smallest antigen epitope, and marks the location of epitope distribution with different colors (Fig. 3).

### Verification of antigenicity of minimal antigenic epitope by TAMV-positive sheep serum

**Fig. 4:**
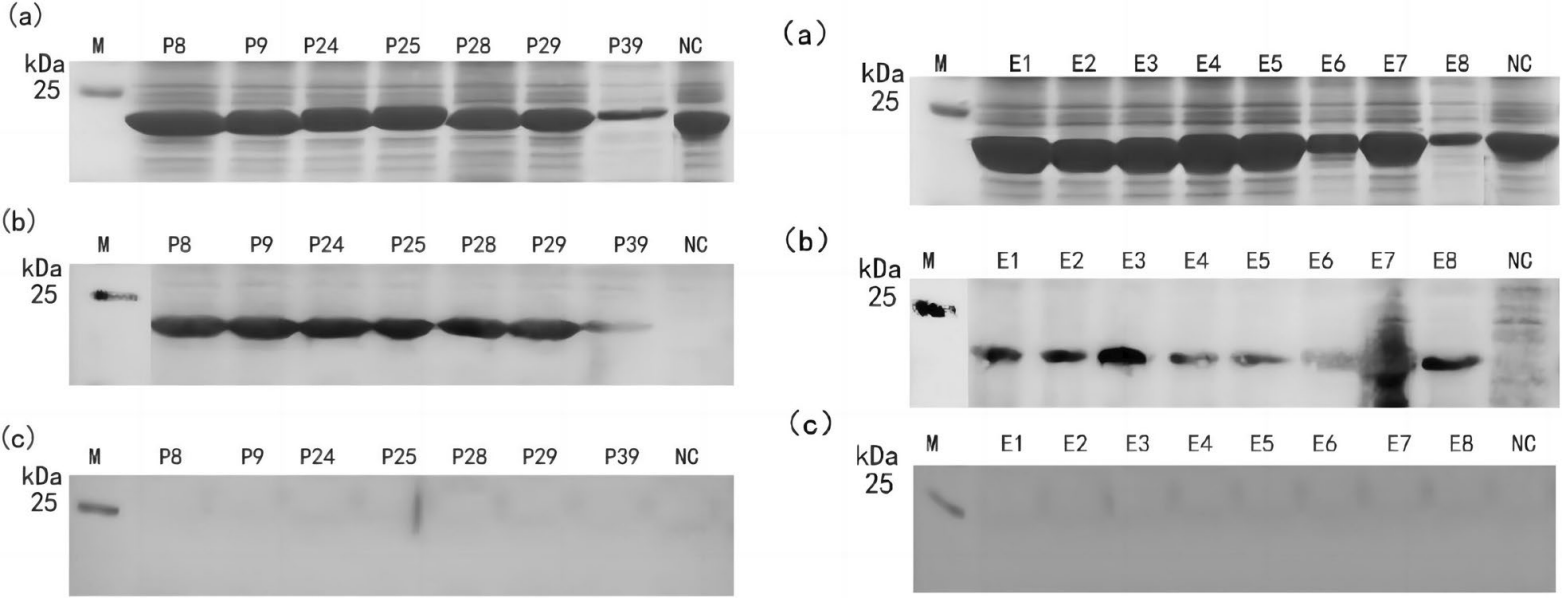
Identification of positive antigenicity of 16-peptide and 8-peptide in sheep serum. **a** SDS-PAGE identification result. **b** Positive serum western blotting detection results. **c** Results of negative serum western blotting detection. *NC* negative control (GST-188 label protein expressed by pXXGST-3 vector), *M* protein marker

The seven positive 16-peptides and eight BCEs identified in this experiment were employed as antigens, and sheep sera collected in Burjin County, Altay, Xinjiang in 2019 were utilized as primary antibodies. The results indicated that seven positive 16-peptides and eight BCEs (Fig. 4) were accurately expressed and could be recognized by positive sheep sera, but not by negative sera.

### Construction and identification of recombinant plasmid

**Fig. 5:**
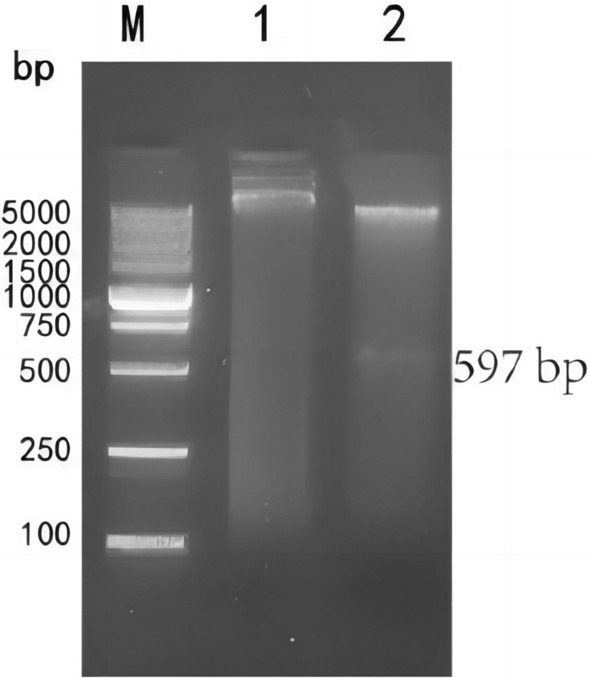
Identification of recombinant plasmid pET-28a-Gn-MEPX_2_ by double enzyme digestion. M DNA maker DL5000; 1 pET-28a-Gn-MEPX_2_ before double enzyme digestion; 2 pET-28a-Gn-MEPX_2_ after double enzyme digestion.

The recombinant plasmid pET-28a-Gn-MEPX_2_ was extracted and digested with *Bam*H I and *Sal* I restriction endonucleases, and the target band emerged at the position of 597 bp (Fig. 5). Further sequencing revealed that the recombinant plasmid was constructed successfully.

### Induced expression and purification of fusion multiepitope peptide (r-Gn-MEPX_2_)

**Fig. 6:**
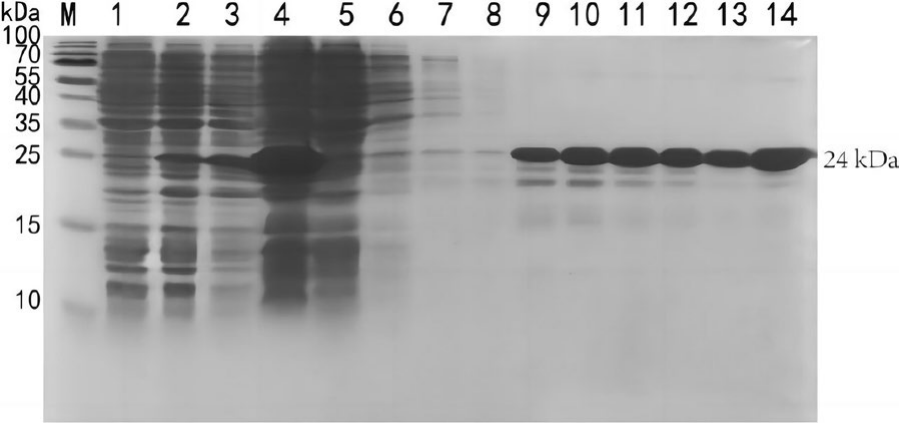
Induced expression and purification of fused multiepitope peptides. (1, 2) pET-28a-Gn-MEPX_2_/Rosetta (DE3) before and after induced expression. (3, 4) Supernatant and precipitation. (5) Flow-through samples; 6–14 20, 20, 20, 100, 150, 200, 200, 200, and 500 mmol/L imidazole eluted protein samples. *M* protein marker

The results of SDS-PAGE electrophoresis indicated that r-Gn-MEPX_2_ was successfully expressed after IPTG induction, and the target band emerged at the location of 24 kDa (Fig. 6), which was in accordance with the expected size. The results of ultrasonic fragmentation revealed that there was a target protein in both the supernatant and the inclusion body, and the protein was mainly expressed in the inclusion body. After elution with different concentrations of imidazole, it was found that the elution effect was better when the concentration of imidazole was 200 mmol/L and 500 mmol/L.

### Antigenicity identification of fusion multiepitope peptide (r-Gn-MEPX_2_)

**Fig. 7:**
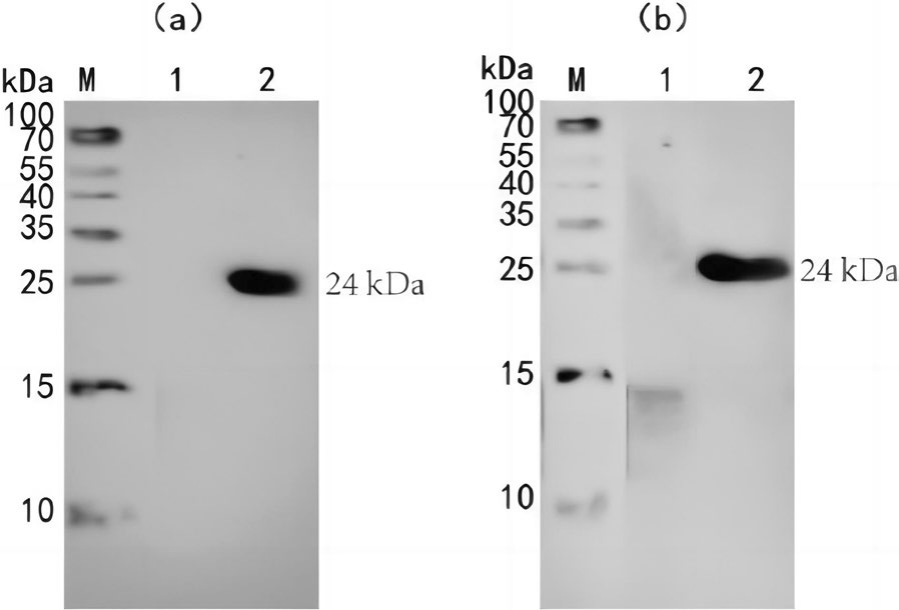
Antigenicity identification of fused multiepitope peptide (r-Gn-MEPX_2_). **a** Mouse anti His-tag mAb was used as a primary antibody to identify the antigenicity of r-Gn-MEPX_2_; **b** The rabbit anti-fusion protein Gn polyclonal antibody was used as the primary antibody to identify the antigenicity of r-Gn-MEPX_2_. *M* protein marker. (1) Before induced expression of r-Gn-MEPX_2_. (2) After induced expression of r-Gn-MEPX_2_

The purified fusion multiepitope peptide was detected by HRP-mouse anti-His monoclonal antibody and rabbit anti-Gn protein polyclonal antibody. Western blotting results demonstrated that the purified fusion multiepitope peptide had antigenicity at the 24 kDa site (Fig. 7), suggesting that the purified fusion multiepitope peptide could be recognized by HRP-mouse anti-His monoclonal antibody (Fig. 7a) and rabbit anti-Gn protein polyclonal antibody (Fig. 7b). It can be utilized to establish an indirect ELISA detection method in the later stage.

### Optimization of working conditions of indirect ELISA detection method based on r-Gn-MEPX_2_

**Fig. 8:**
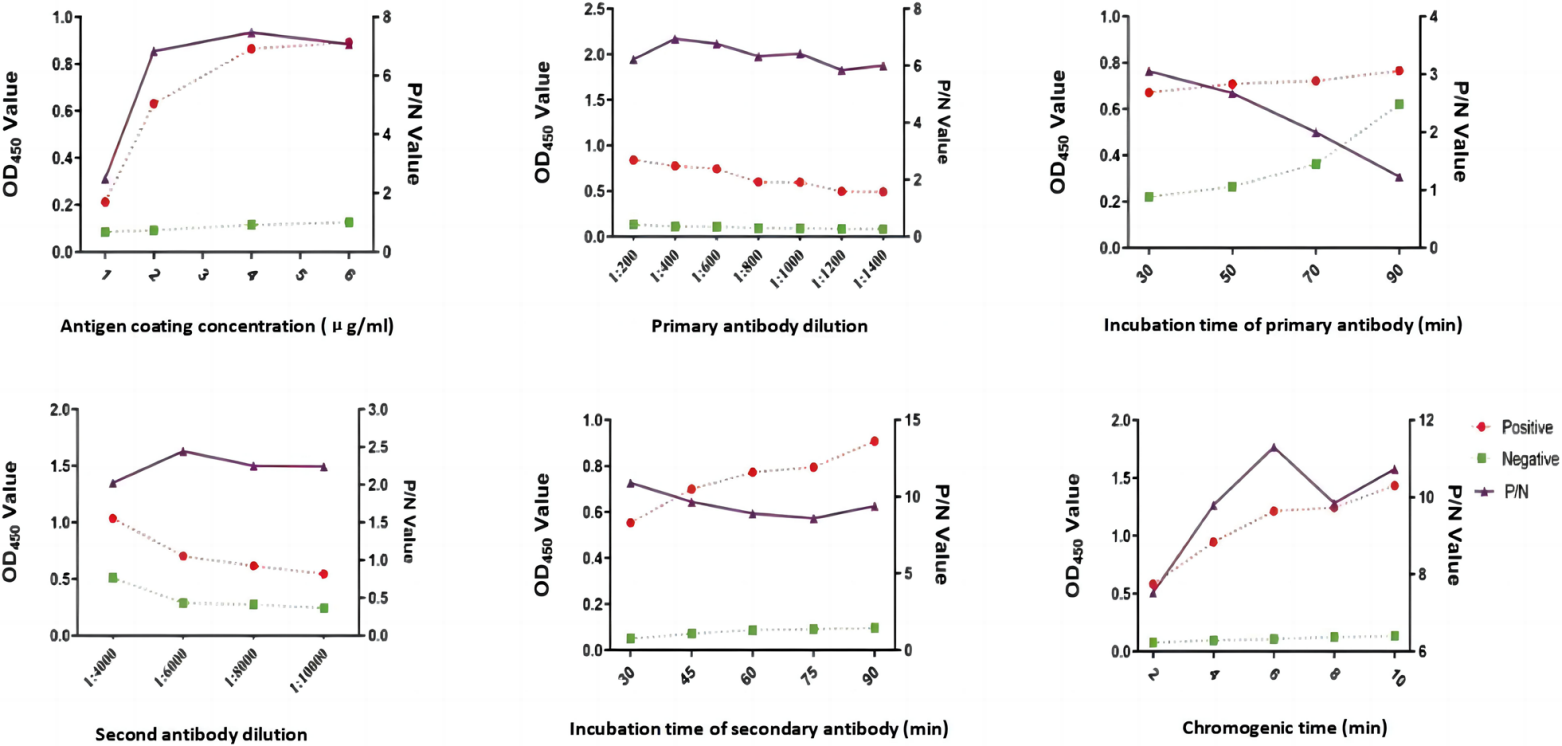
Condition optimization of indirect ELISA detection method based on r-Gn-MEPX_2_

After optimization, the indirect ELISA detection method based on r-Gn-MEPX_2_ reached the maximum P/N value when the antigen concentration was 4 μg/mL, the dilution of the primary antibody was 1:400, the incubation time of the primary antibody was 30 min, the dilution of the secondary antibody was 1:6000, the incubation time of the secondary antibody was 30 min, and the chromogenic time was 6 min (Fig. 8).

### Accuracy of indirect ELISA detection method based on r-Gn-MEPX_2_

**Fig. 9:**
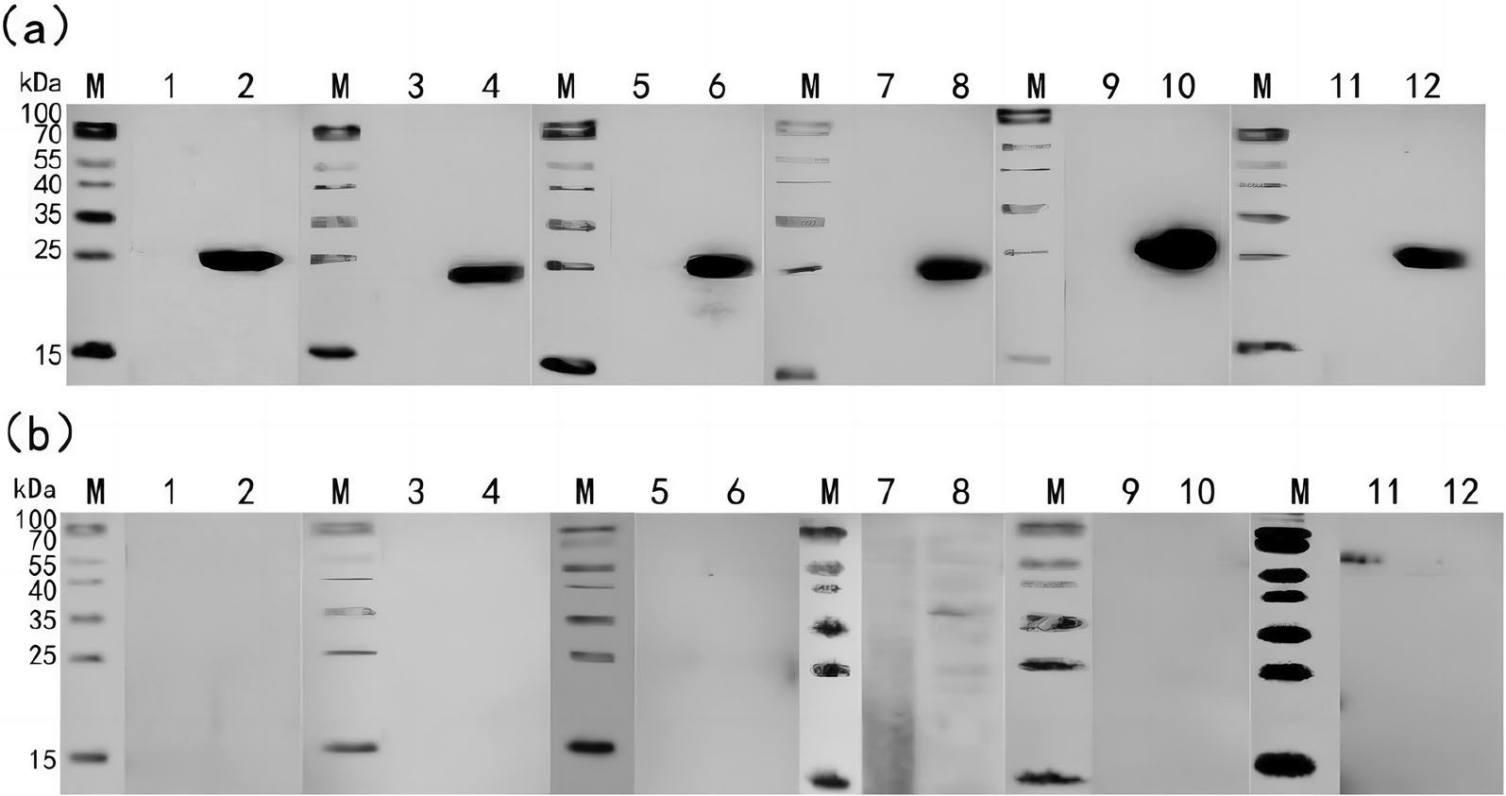
Western blotting verifies the accuracy of indirect ELISA detection based on r-Gn-MEPX_2_. **a** Western blotting of r-Gn-MEPX_2_ and the positive serum screened by indirect ELISA. **b** Western blotting of r-Gn-MEPX_2_ and negative serum screened by indirect ELISA. *M* protein marker; 1, 3, 5, 7, 9, 11 blank control (5% BSA unrelated protein); 2, 4, 6, 8, 10, 12 purified r-Gn-MEPX_2_

With r-Gn-MEPX_2_ as antigen, six sera of TAMV-positive and 6 seronegative animals in Xinjiang were screened by indirect ELISA detection method based on r-Gn-MEPX_2_. The accuracy of the established indirect ELISA detection method was verified by western blotting. All six positive animal sera presented immunoblotting reaction at the site of approximately 24 kDa (Fig. 9a), while six negative sera showed no target band (Fig. 9b), which was consistent with the results of the established indirect ELISA assay.

### Sensitivity of indirect ELISA detection method based on r-Gn-MEPX_2_

The positive and negative sera of TAMV were diluted and detected by the established indirect ELISA detection method based on r-Gn-MEPX_2_. P/N > 2.1 is deemed positive, suggesting that the detection method is efficacious at this dilution. The results demonstrate that the serum dilution up to 1:1600 can also be detected (Table 1), indicating that the detection method exhibits high sensitivity.

**Table 1:** Sensitivity test results of indirect ELISA based on r-Gn-MEPX_2_

Dilution degree	1:100	1:200	1:400	1:800	1:1600	1:3200
P (OD_450nm_)	1.847	1.733	1.434	1.05	0.745	0.499
N (OD_450nm_)	0.685	0.578	0.502	0.432	0.318	0.266
Detection result	+	+	+	+	+	−

‘+’ represents positive

‘−’ represents negative

### Specificity of indirect ELISA detection based on r-Gn-MEPX_2_

The established indirect ELISA detection method was employed to detect the serum of four kinds of virus-related polyclonal antibodies in order to evaluate the specificity of this method. The results indicated that only the positive serum of TAMV was positive and the others were negative (Table 2), indicating that the method is specific in application.

**Table 2:** Specificity of indirect ELISA based on r-Gn-MEPX_2_

Serum	TAMV	GTV	CCHFV	SFTSV	RBDV	Negative control
OD_450nm_	1.507	0.205	0.141	0.196	0.165	0.117
Detection result	+	−	−	−	−	−

‘+’ represents positive; ‘−’ represents negative

*GTV* Guertu virus; *SFTSV* Severe fever with thrombocytopenia syndrome virus; *RBDV* Rhabdo virus; *CCHFV* Crimean-Congo hemorrhagic fever virus

### The results of indirect ELISA detection in animal serum

Indirect ELISA detection method was employed to detect 327 animal sera in Xinjiang. A total of 15 anti-TAMV Gn protein IgG-positive sera were identified. The total positive rate was 4.59%.

## Discussion

TAMV pertains to the genus *Orthoroviridae*, which can induce fever and other symptoms upon infection [22]. As the virus is a novel one, there are scarce studies on it. Currently, an ELISA detection method based on TAMV GP protein [23] and a rapid detection method of real-time reverse-transcription PCR based on TaqMan [24] have been established. TAMV GC and Gn proteins [25] have been successfully expressed in prokaryotic and eukaryotic systems, and corresponding polyclonal antibodies have been prepared. In this study, the enhanced biosynthetic peptide method [26] was utilized to screen the fine epitopes of TAMV Gn fragments, with the aim of establishing an efficient and rapid detection method.

Epitopes, also known as antigenic determinants, are critical antigenic components of viral structural proteins that can be recognized by the immune system, including antibodies, B cells, and T cells. The activation of memory B cells relies on the recognition of these epitopes, which play a pivotal role in the development of vaccines targeting extracellular pathogens. Therefore, this study is aimed at detecting fine antigenic epitopes with immunogenicity on the full fragment and employs flexible peptide chains. The flexibility of the peptide enables the conformation to change, which facilitates binding and recognition, significantly enhancing the ability of the fragment to be recognized by the immune system and reducing the influence of conformation changes on the recognition ability.

In this study, seven positive 16-peptides were screened, and their good antigenicity was verified by western blotting. Subsequently, the seven positive 16-peptides were truncated into 8-peptides overlapping seven amino acids, and ultimately eight fine antigenic epitopes were identified. The good antigenicity of these epitopes was also confirmed by western blotting. After analysis, it was discovered that they were all situated on the surface of the three-dimensional structure of glycoprotein Gn and were highly conserved among different TAMV strains. The genes of the eight identified fine epitopes were concatenated and double-copied and expressed in the prokaryotic system to form the fusion multiepitope peptide (r-Gn-MEPX_2_). The prokaryotic expression system possesses the advantages of relatively simple construction steps, high protein expression, and a short expression cycle [27]. In this study, the prokaryotic expression system pET-28a-Gn-MEPX_2_ was transformed into *E. coli* BL21 (DE3) competent cells to induce the expression of recombinant protein. An indirect ELISA detection method based on the fused multiepitope peptides was established.

By employing the established ELISA detection method, six positive and six negative sera were examined, all of which were detected while the negative ones were not. The positive and negative sera of TAMV were diluted in multiple ratios. When the maximum dilution of the serum was 1:1600, it could still be detected.

Subsequently, the established indirect ELISA detection method was employed to detect the sera of four types of virus-related polyclonal antibodies, and only TAMV could be identified. This method eliminated the influence of weakly antigenic fragments, thereby having high sensitivity, accuracy, and specificity. The ELISA detection method based on r-Gn-MEPX_2_ established in this study furnishes an effective and alternative serological detection technique for investigating the prevalence of TAMV in the future.

However, in this study, we merely tested serum samples from the Xinjiang region, and the sample area is subject to certain limitations. In addition, the fusion multiepitope peptide (r-Gn-MEPX_2_) is an inclusion body protein, which encounters certain difficulties during the purification and preservation processes. These will be the issues that need to be addressed in the future.

## Conclusions

The genes of eight identified elaborate epitopes were concatenated and double copies were expressed in the prokaryotic system to form the fused multiepitope peptide (r-Gn-MEPX_2_). An indirect ELISA detection method based on the fused multiepitope peptide was established; this method was used to detect animal sera in some areas of Xinjiang, and the results indicate that the method has high sensitivity, accuracy, and specificity. A total of 327 animal sera in Xinjiang were detected, and the positive rate was 4.59%.

## Data Availability

No datasets were generated or analyzed during the current study.
